# Applying a mixed methods design to test saturation for qualitative data in health outcomes research

**DOI:** 10.1371/journal.pone.0234898

**Published:** 2020-06-19

**Authors:** Fatoumata Fofana, Pat Bazeley, Antoine Regnault

**Affiliations:** 1 Patient-Centered Outcomes, ICON plc, Lyon, France; 2 UMR INSERM 1246, SPHERE « methodS in Patients-centered outcomes and HEalth ResEarch », University of Nantes, University of Tours, Nantes, France; 3 Western Sydney University, Sydney, Australia; 4 Modus Outcomes, Lyon, France; The MetroHealth System and Case Western Reserve University, UNITED STATES

## Abstract

Saturation, a core concept in qualitative research, suggests when data collection might end. It is reached when no new relevant information emerges with additional interviews. The aim of this research was to explore whether a mixed methods design could contribute to the demonstration of saturation. Firstly, saturation was conceptualized mathematically using set theory. Secondly, a conversion mixed design was conducted: a set of codes derived from qualitative interviews were quantitized and analyzed using partial least squares (PLS) regression to document whether saturation was reached. A qualitative study conducted by other researchers prior to this work (i.e. none of the present authors was involved in this study) was used to test saturation using PLS regression. This illustrative qualitative study aimed to investigate the impact of *Clostridium difficile* infection (CDI) on nurses’ work in the hospital and the results were published elsewhere (Guillemin et al. 2015). Semi-structured interviews were conducted with 12 nurses. Saturation was characterized by the cumulative percentage of variability accounted for by PLS factors. After 12 interviews, this percentage was 51% which suggests that saturation was achieved at least on main themes. Two main themes identifying similarities in the experience of nurses caring for patients with CDI were identified: *Organization/Coordination of the working day* and *Time-consuming work*. Although dependent on the coding of qualitative data, PLS regression of quantitized data from qualitative interviews generated useful information for the determination of saturation.

## Introduction

### Background

The determination of an adequate sample size to appropriately address a research question is a fundamental aspect of the data collection process in any context, qualitative or quantitative. In quantitative research, adequate sample size is determined using an inferential statistical framework, based on assumptions on type I and II error control. In qualitative research, the concept of saturation, also known as ‘data saturation’, has been proposed as the solution to determine an adequate sample size. Morse defined saturation as “data adequacy” which means that saturation is reached when data collected is sufficient to cover the themes of interest and that collecting further data will not bring new relevant information [1]. Saturation is commonly documented through a saturation grid which consists of a simple report of the occurrence of a theme (displayed in a row) elicited during each interview (displayed in a column) in a tabular format [2]. In the grid, saturation is considered reached when the column for the current interview is empty, suggesting that no new themes have been elicited [2]. Over the last few years, there has been a growing interest in methods for determining sample size in qualitative research and researchers have proposed various approaches to operationalize saturation or to come up with an adequate sample size to reach saturation [1, 3–15]. Some have proposed quantitative methods to address the saturation question. Tran et al. used a mathematical model developed for ecological studies to extrapolate the accumulation of themes and to help researchers determine the point of data saturation in studies with open-ended questions [13]. van Rijnsoever used data resampling with a simulation approach to explore the sample size required to reach theoretical saturation [15]. Lowe et al. proposed a mathematical model for both quantifying saturation (by describing the progress of theme acquisition) and estimating the number of observations required to achieve a specified level of saturation [6].

Mixed methods research (MMR) can be defined as “research in which the investigator collects and analyzes data, integrates the findings, and draws inferences using both qualitative and quantitative approaches or methods in a single study or program of inquiry”[16]. MMR has been increasingly used in health outcomes research over the past years for different purposes [17]: for example, to explore the benefit-risk balance from the patient perspective in rare diseases [18]; to generate information to support the interpretation of patient-reported outcome (PRO) scores [19, 20]; to capture the patient’s and family’s voice in natural history studies for orphan drug development [21]; or to create fit-for-purpose PRO measures [22, 23]. Besides, MMR is recognized as a useful method to generate patient-centered evidence by regulatory agencies [24].

MMR offers a variety of study designs that are used depending on the research question. In the health outcomes field, one of the most common mixed methods designs is the convergent parallel design [25, 26]. The convergent parallel design is used when the researcher wants to conduct study conceptualization (i.e. defining the research question), data collection, data analysis from both qualitative and quantitative strands independently but concomitantly [25, 27]. Ultimately, the two sets of results are merged and then interpreted to draw an overall conclusion. In our research, we used a convergent parallel design including data transformation. This type of design is called a conversion mixed design [28], a multistrand parallel design in which mixing of qualitative and quantitative approaches occurs when one type of data is converted (qualitized or quantitized) and then analyzed both qualitatively and quantitatively [29]. Quantitizing coded qualitative data is usually achieved by considering the codes as variables, and counting either presence/absence for each case, or the number of occurrences for each case.

The objective of this study was to explore whether a conversion mixed design using a quantitative approach could contribute to the determination of saturation in qualitative research, based on a new analysis of data from an existing qualitative study.

### Presentation of the illustrative qualitative study

Existing data from a qualitative study were used to address our objective. This qualitative study aimed to investigate the impact of the *Clostridium difficile* infection (CDI) on nurses’ work in the hospital. *Clostridium difficile* is a common source of antibiotic-associated diarrhea and leads to nosocomial infection [30]. Semi-structured interviews of 12 nurses (six in France and six in the US) were carried out with the support of an interview guide. Nurses included in the study worked in an intensive care ward in the hospital or in a nursing home and had managed up to 60 cases of CDI in the year prior to the interview [30]. The qualitative researchers identified 67 themes spontaneously elicited from nurses during their interviews (S1 Table). In their published paper, the qualitative researchers followed the principle of data saturation to justify adequate sample size, using the following definition of saturation: “Saturation is considered to have been attained for a concept when no new concepts or relevant information emerges with additional interviews” [30].

## Materials and methods

### Mathematical conceptualization of saturation

**Fig 1:**
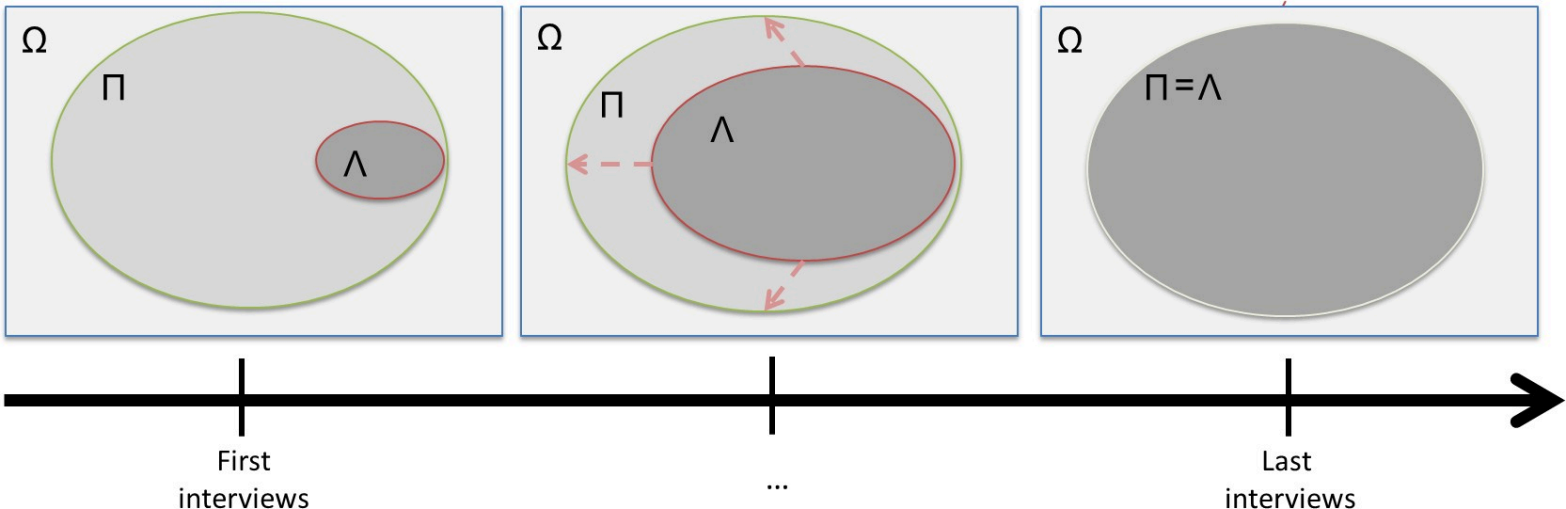
Schematic modeling of saturation in qualitative research using set theory. Ω = Universe of themes; Π = Set of targeted themes; Λ = Set of relevant themes elicited during interviews.

We started by conceptualizing saturation in a quantitative framework. For this purpose, the focus was shifted from sample size to number of themes and set theory was used (Fig 1). It was assumed that a universe of themes exists (Ω) in which there is a subset of themes of interest regarding the research question (Π). During the interviews, there is a set of relevant themes that are elicited from interviewees (Λ), which grows as more interviews are conducted. In this framework, saturation is reached when Λ = Π. In this quantitative framework, a theme is considered relevant if it brings a meaningful contribution in the answer to the research question.

### Application of the conversion mixed design to the saturation question

**Fig 2:**
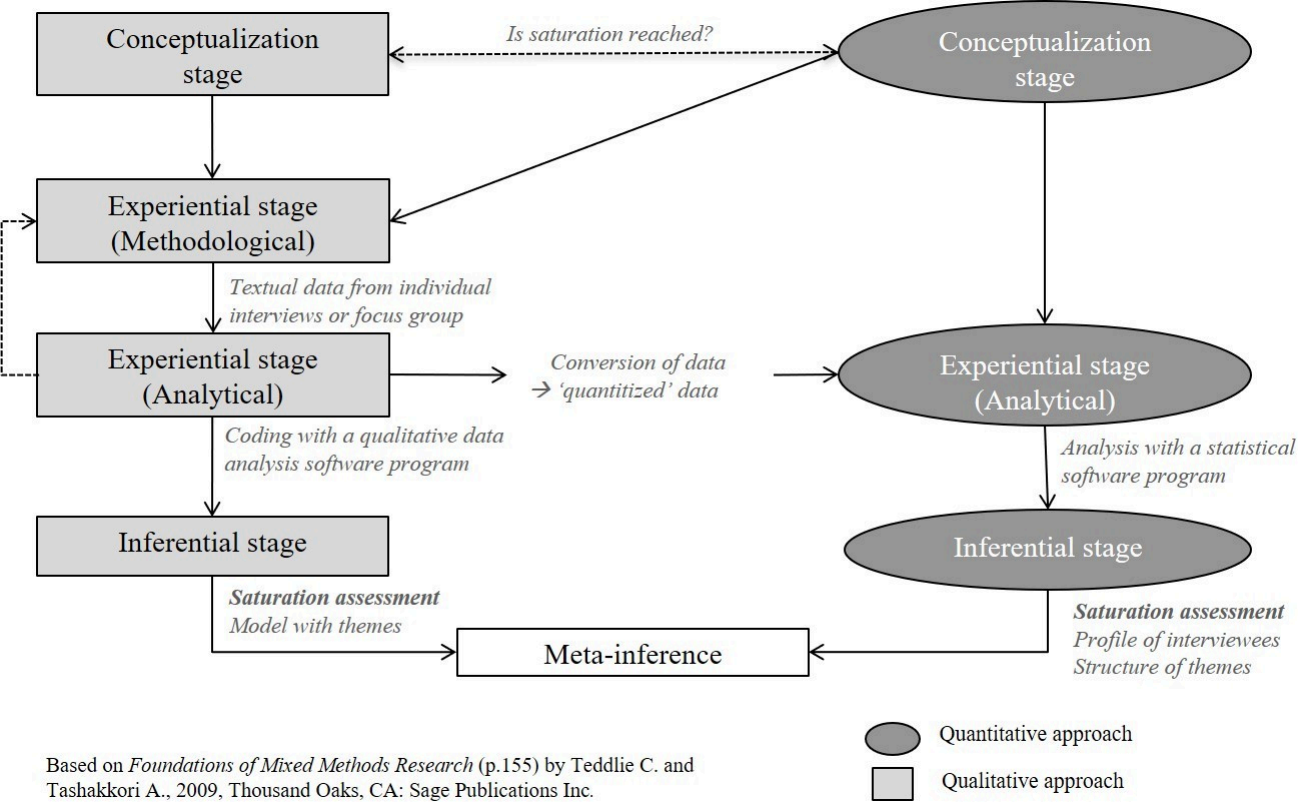
Graphic illustration of the conversion mixed design adapted to the question of saturation. The qualitative strand was completed prior to and independently of the present work.

The schematic illustration of the conversion mixed design used for our research question is displayed in Fig 2. The qualitative and quantitative approaches are presented in parallel. The common research question that both strands are addressing is: “Is saturation reached?”

#### Qualitative strand for investigating saturation

The qualitative strand that was used in the Guillemin et al. (2015) study on which this one is based followed a standard process to address the question of saturation [30]. Qualitative data were collected using the sequential order of the interviews and analyzed thematically, i.e. coded by the qualitative researchers, with saturation being declared when no new themes were forthcoming. Personal communication with the first author of the paper (Dr. Guillemin), revealed that saturation had been demonstrated using a saturation grid. The coding process of the qualitative interviews was undertaken with the assistance of a qualitative data analysis software program (Atlas.ti).

#### Quantitative strand for investigating saturation

In our study, existing qualitative data were quantitized to be used in the quantitative strand. Codes for each case were converted into numerical data with the help of the qualitative data analysis software program [31, 32]. The data conversion consisted in counting the codes used for each theme elicited during the interviews, recording the number of times each was mentioned for each interview. This database was then used for the statistical analysis. In our study of saturation, partial least squares (PLS) regression was applied to allow the mathematical conceptualization of saturation as defined above to be operationalized. PLS regression is a statistical technique that models relationships between a set of dependent variables (Y) and a set of independent variables or predictors (X) [33–36]. PLS regression is known to be robust to both small sample size and high collinearity among the independent variables [37, 38], which are often a problem when using quantitized data from interviews. The principle of PLS regression is to extract from X and Y a pair of latent components called PLS factors denoted (t,u), for which their covariance is maximal (t is orthogonal) [33]. PLS regression is sometimes described as a combination of principal component analysis (PCA) and linear multiple regression [33]. Several outputs are available for interpretation from PLS regression. First, the cumulative percentage of variation in themes explained by PLS factors is an indicator comprised between 0 and 100 that can be interpreted as an R-square. Second, the correlation-loading plot is a graphical representation of the space defined by PLS factors in which can be presented both the observations and the variables. It also displays the correlation between the input X- and Y-variables and the PLS factors. [34, 35]. Third, the PLS t_i_/u_i_ score plot is the projection of the observations in the X-space (score t_i_) and in the Y-space (score u_i_) according to the PLS factor i. It shows how well the Y-space correlates with the X-space [34].

For the purpose of investigating saturation with PLS regression, the interviews were split into two blocks of interviews, categorized sequentially: the first block is composed of a large part of the interviews conducted (i.e. the first 75% interviews) and the second block comprised the remaining interviews. The objective is to estimate the difference between the set of themes of interest regarding the research question (Π) and the set of relevant themes elicited during the interviews (Λ) by the difference between the first interviews and the last interviews (Fig 1). If the content of the first interviews explains largely the content of the last interviews, then this means that only marginal new additional information (themes) emerged from the last interviews and that saturation was achieved. This PLS regression model can be written as follows:

$$(X_{j+1}\ldots X_{n})=(X_{1}\ldots X_{j})\;B_{PLS}+E$$

Where:

▪ X_j_ corresponds to the vector of the number of times each theme is coded in the j-th interview
▪ B_PLS_ is the vector of regression coefficients
▪ E is the matrix of residuals

This model expresses the regression of the themes elicited in the set of last interviews (set of X_j+1_-X_n_) by the themes elicited in the set of the first interviews (X_1_-X_j_). The PLS model was performed using SIMCA software (formerly SIMCA-P) [39].

### Meta-inference phase

The final stage in the conversion mixed design, as in any parallel mixed methods design, is the meta-inference based on combining or comparing the results of the two strands: in this case, whether the results provided by the quantitative strand regarding saturation are supportive to those obtained from the qualitative strand.

## Results

### Qualitative strand results

The authors of the qualitative analyses, which were reported earlier elsewhere, considered that saturation had been reached and that an understanding of how CDI affected nurses’ work was comprehensively captured at the end of the 12 nurse interviews (NIs) [30].

### Quantitative strand results

PLS regression was performed to link the block of the themes elicited in the last three NIs to the block of the themes elicited in the first nine NIs. The PLS factors summarizing all the themes from the first nine NIs explained 51% of the variability of the themes from the last three NIs (Table 1). Only the first PLS factor contributed at a significant level according to cross validation for both the block of themes of the first interviews and last interviews: it explained 34% of the variability of the first nine NIs and 39% of the variability of the last three NIs. For this first PLS factor, the underlying structure covering the themes elicited in the first set of NIs was predicting the underlying structure of the last NIs: this supports the conclusion that saturation was reached, at least on the main themes covered by that factor (see details below). A main theme was defined as a theme that was the most frequently elicited by nurses during their interviews and emerged as critical for nurses. Each of the other PLS factors also contributed roughly equally to the explanation of the total variability of the first nine NIs and marginally the last three NIs.

**Table 1:** Cumulative percentage of variation of the first nine nurse interviews and the last three nurse interviews taken into account by PLS factor (N = 67).

Number of PLS factors	Cumulative percentage of variation taken into account by PLS factor		
	First 9 nurse interviews (= independent variables)	Last 3 nurse interviews (= dependent variables)	Significance*
1	33.57	38.66	S
2	41.04	46.10	NS
3	56.14	48.29	NS
4	62.78	50.01	NS
5	73.18	50.45	NS
6	80.88	50.73	NS
7	90.09	50.84	NS
8	94.94	50.92	NS
9	100.00	50.93	NS

*Significance was determined by cross validation at α = 0.05; S = significant; NS = non-significant

### Combination of qualitative and quantitative strand results (Meta-inference)

Both quantitative and qualitative results suggested that saturation was reached in this illustrative qualitative study. PLS regression showed that saturation was reached for at least main themes while qualitative results suggested that saturation had been reached.

### Additional quantitative findings from the PLS regression analysis of the quantitized qualitative data

**Fig 3:**
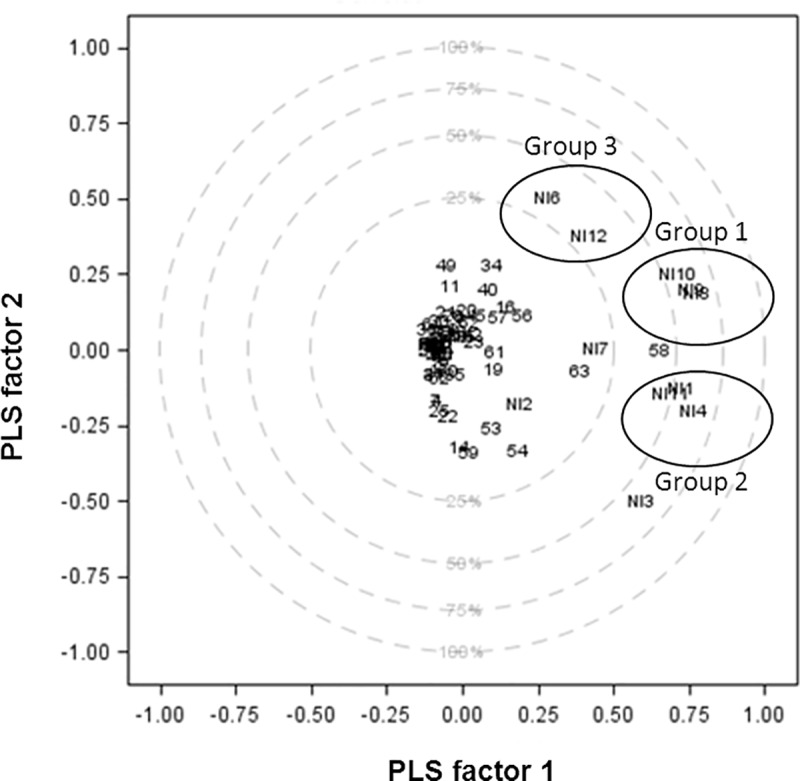
Correlation-loading plot of the relationships between nurse interviews overlaid with the themes in the 2 PLS factors space (N = 67). NI = Nurse Interview; Legend: Outer circle is the unit circle and indicates 100% of explained variance. Inner circle indicates 25% of explained variance. Numbers represents all the themes elicited during nurse interviews.

PLS regression also allowed a more detailed exploration of the structure of the themes and the definition of profiles of the NIs, based on the first two PLS factors that captured most of the variability of the data. The association of the two blocks of NIs was observed using the correlation-loading plot of the two first PLS factors (Fig 3). The graph showed the association between the first nine NIs and the last three NIs: the closer NIs are on this plot, the more similar is their experience when caring for patients with CDI. Three groups of NIs were identified from the correlation-loading plot, which we called Group 1 (representing between 50% and 75% of explained variance), Group 2 (representing between 50% and 75% of explained variance) and Group 3 (representing between 25% and 50% of explained variance) on the graph. Nurses within each of these three groups shared common experience when caring for patients with CDI. Additionally, at least one NI of the last three NIs was present in each of the groups, which reinforces the evidence of saturation.

**Fig 4:**
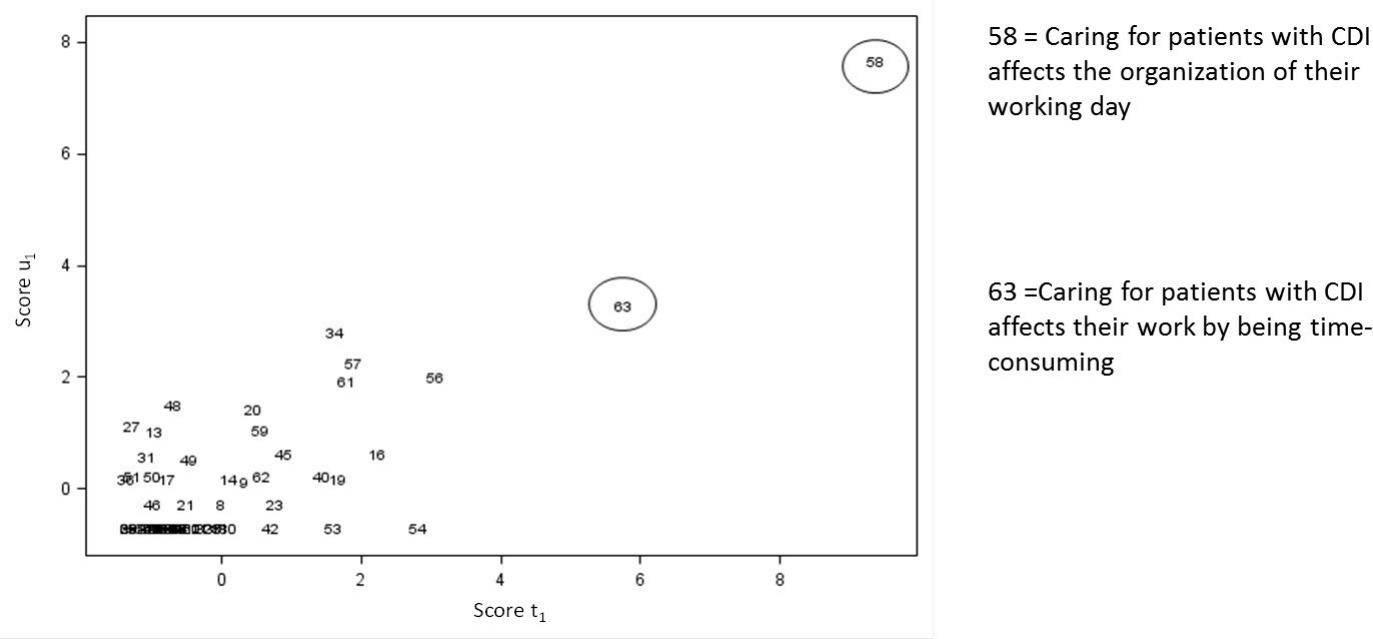
Projection of the themes in the space of the predictor score (t1) and the response variable scores (u1) according to the first PLS factor (N = 67).

**Fig 5:**
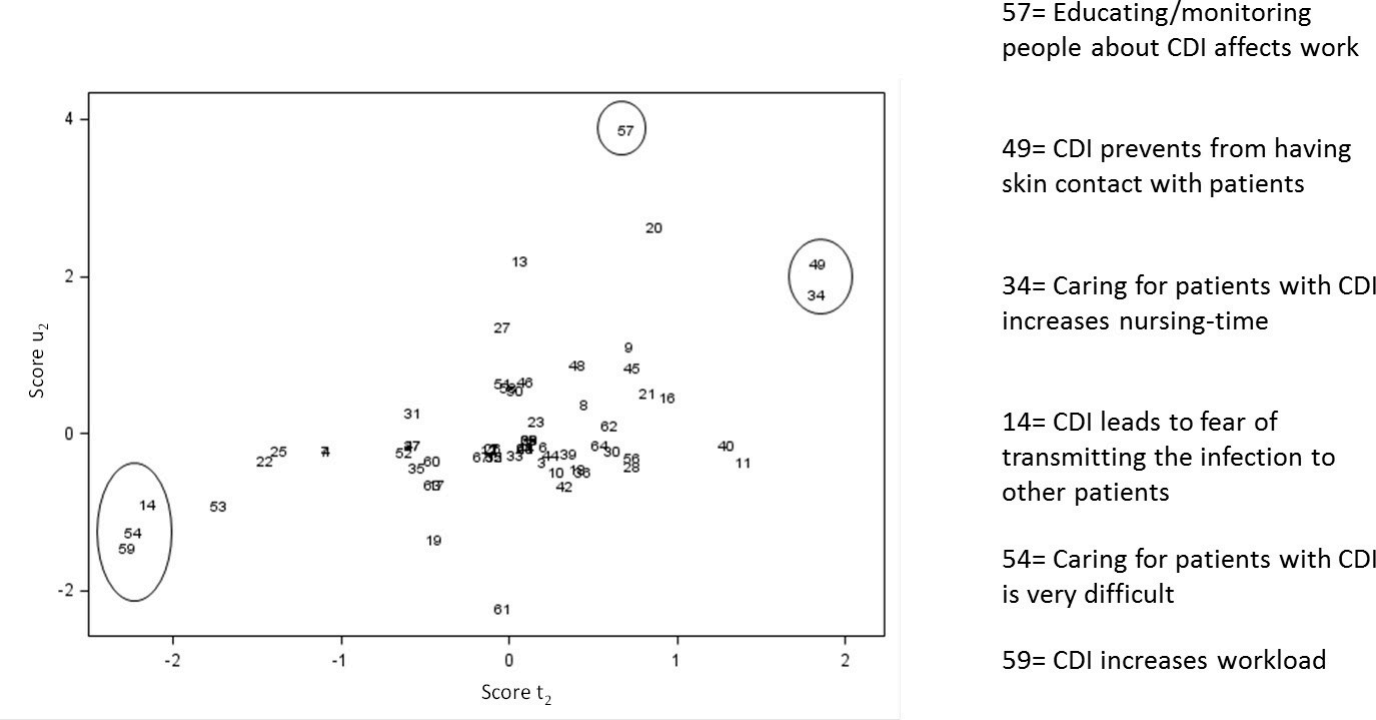
Projection of the themes in the space of the predictor score (t2) and the response variable scores (u2) according to the second PLS factor (N = 67).

The interpretation of PLS regression requires the interpretation of what drives the creation of PLS factors: in our case, what are the themes that were contributing the most to the creation of these factors. This can be represented by the scatter plot of the projections of the themes in the space of PLS factors (Figs 4 and 5). These figures, as a complement to the correlation loading plot, came to the following findings: crucial themes for nurses from Group 1 and Group 2 were that caring for patients with CDI affected the organization of their working day (theme n°58 –Fig 4) and also affected their work by being time-consuming (theme n°63 –Fig 4). NIs from Group 3 emphasized that caring for a patient with CDI increased their nursing-time (theme n°34 –Fig 5) and prevented them from having skin contact with patients (theme n°49 –Fig 5).

## Discussion

The objective of our study was to explore whether a conversion mixed design could contribute to the determination of saturation in qualitative research. In the illustrative qualitative study, results showed that saturation had been reached after 12 interviews [30]. Our quantitative results corroborated in part this conclusion as we showed that saturation was reached for at least the main themes (i.e. themes that were the most frequently elicited across the NIs) in the first nine NIs. PLS regression results showed that the theme-based factors for first nine NIs were able to explain 51% of variability in the last three NIs. By nature, interviews generate rich and complex information and explaining more than half of the between-interview variance in the themes elicited in this context is considered satisfactory. Besides, the first PLS factor, which explained respectively 34% and 39% of the variance of respectively the first NIs and the last three NIs, was significant according to the cross validation test. This shows that this solid, replicable factor covered a similar proportion of the variance in the two sets, which reinforces the indication of saturation being reached. Finally, in the correlation-loading plot, none of the three last interviews was an outlier compared to other interviews (which would have signified completely different interview content). The concordance of the results regarding saturation between the statistician and the qualitative researcher in this study strengthens our confidence in saturation being reached. While there are no absolute standards as to what constitutes an acceptable level of explanation of variance when using multivariate data analyses, 51% would generally be considered acceptable, especially as the first factor was found to be significant according to the cross validation test. We invite researchers who are willing to conduct similar work to compare their results with ours and we are hoping that accumulated evidence will help set standards for statistical analyses on qualitative data.

Since Morse pointed out that “there are no published guidelines or tests of adequacy for estimating the sample size required to reach saturation” [1], several researchers have attempted to address the gap in guidelines on how to operationalize saturation, as noted earlier. On one hand, these researchers included mainly theoretical considerations regarding the operationalization of saturation and proposed a benchmark to determine a priori when saturation might be reached [3, 4, 14]. Judgements about repetition of themes do not offer true operational solutions to qualitative researchers when they have to make a decision on saturation while quantitative approaches may lack generalizability as they may be context-specific (dependent on the research question or the targeted population for example) or based on strong assumptions that may not hold in actual qualitative research settings [5, 11]. Our approach differs in that it offers a process that can be applied by a qualitative researcher to their own data to provide guiding information on when saturation is achieved. Other researchers who applied a quantitative approach to the question of saturation either used techniques that required a large number of interviews (more than 100) [13, 15] or techniques that assumed statistical independence of observations (i.e., across interviews) [6], neither of which are reasonable in most qualitative research contexts.

Our solution based on a mathematical conceptualization of saturation using test theory and quantitative multivariate analysis of quantitized data used frequencies (i.e. numerical data) of the themes as input to quantitative analyses instead of binary occurrence (Yes/No). These frequencies were obtained through conversion using qualitative research software outputs to capture the frequency of occurrence of the theme while also providing information about how meaningful the theme was for the interviewee. This conversion of qualitative data into quantitized data does not come without assumptions: the frequency of a theme is seen as indicative of the importance of the theme for the interviewee but reduces the meaning of the theme into a fixed and single dimensional notion [40]. We acknowledge that this choice can be challenged: 1) the frequency of the theme elicited may be driven by the course of the discussion and the speaking style of the interviewee, and 2) the use of frequencies distances from the typical method of identifying saturation by the qualitative researcher. The possibility to adapt the quantitative multivariate analysis, especially the PLS regression, to analyze occurrence instead of frequency of themes could be further investigated.

Besides of the type of data used, other factors influence when the saturation has been reached. These include the research question which is of extreme importance, the interviewee characteristics, the relevant information obtained from each interviewee, the sampling strategy, the qualitative method used and how the data have been analyzed qualitatively [41]. In particular, the level of detail used in the coding exercise (“granularity” or unit of information) is at the core of the qualitative data conversion and is therefore critical for the interpretation of the results of the quantitative analysis. The smaller the unit of information (i.e. being captured by the code, with consequently a greater number of codes being used), the more difficult the demonstration of saturation will be. In addition, the initial coding strategy may be changed while the interviews happen, and some themes of special interest can be more developed, with always finer details and always more specific codes. As quantitative methods use the codes as input, their outputs, such as the percentage of variance explained for example, will be impacted by the granularity of the codes. However, as statistical methods such as PLS regression are designed to summarize information in large datasets, they might allow disentangling true information from noise and therefore be able to reliably inform decision on saturation even when the granularity of codes is small.

Our conceptualization of saturation requires different sets of information. For its operationalization, these sets need to be estimated. For this purpose, we split the data from the interviews into two blocks: the first three quarters of the interviews (i.e. the first nine NIs) constituted the first block and was presumed to represent the knowledge established by the research; the second block (i.e. the last three NIs) was constituted of the remaining quarter of the interviews and potentially represented new information. This split was done using the chronological order of interviews because the common sampling and analytical approach in qualitative research allows for emergence of new themes and the possibility that the coding may change over time, building on the experience of the qualitative researcher. This therefore meant that the most stringent test possible regarding the potential for differences in themes for the two groups was applied. Also, it was decided to use a group of interviews to characterize the new information and not a single interview (i.e. the very last one) because a single interview might be a complete outlier. Other specifications of our proposed approach are possible (e.g. different splits into interview blocks through an iterative process) and should be explored to determine the most appropriate way to provide supportive evidence on saturation.

For the quantitative strand of our MMR, PLS regression allowed our conceptualization of saturation to be operationalized, but it also provided useful additional information on the qualitative data by identifying interviewee profiles based on the themes they expressed. PLS regression features provided a detailed typology of NIs and the pattern of themes for both first and last NIs. For example, the various PLS outputs indicate that the two NIs from Group 1 (NI8 and NI9) and one nurse from Group 2 (NI10) were similar, despite being in different groups, because each expressed the theme n°58 (caring for patients with CDI affected the organization of their working day) as something critical to them during their interview.

We focused on qualitative studies using individual interviews for data collection and thematic analysis for analytical methodologies [30]. These are the typical features of a qualitative study in the health outcomes field. Further investigation will be needed to investigate whether our research can be applied in different qualitative research settings, with different qualitative data collection methods (e.g., focus groups, observations, logs, audiovisual material) or different qualitative analytical methodologies (e.g., grounded theory, phenomenology).

Finally, our research provides an application of MMR in the health outcomes field. We demonstrated how a conversion mixed design can provide additional insight on saturation in qualitative research. This expands the toolbox of MMR solutions to address health outcomes research questions. This example, in which a quantitative technique helps inform a typical qualitative question, also shows that qualitative and quantitative researchers can benefit from working closely together and comparing their perspectives. Doing so can lead to innovative solutions for the questions they routinely face in their research.

## Conclusion

Our proposed conversion mixed design, applying PLS regression to data collected in a qualitative study, operationalizes a formal mathematical conceptualization of the core concept of saturation in qualitative research and allows additional supportive, quantitative evidence on saturation to be generated. In our case study, not only did our MMR approach strengthen the demonstration of saturation, it also provided useful substantial information on the research question. Hence, this application of conversion mixed design opens interesting perspectives for the exploration of data generated by qualitative research in the health outcomes field.

## Supporting information

S1 Table Themes elicited from the nurse interviews and used in the PLS regression analysis. NI = Nurse interview; Cdiff = Clostridium difficile infection.
(XLSX)

S1 Appendix Quick guide to PLS regression model using SIMCA software. (DOCX)

## List of abbreviations used

CDI: *Clostridium difficile* infection
MMR: Mixed Methods Research
NI: Nurse Interview
PLS: Partial Least Squares
PRO: Patient-Reported Outcomes
