# Supplementary material for: Applying a mixed methods design to test saturation for qualitative data in health outcomes research
Source: PLoS One. 2020 Jun 19;15(6):e0234898. doi: 10.1371/journal.pone.0234898 (PMC7304618; doi:10.1371/journal.pone.0234898)
Supplement: S1 Appendix — (DOCX) [file pone.0234898.s002.docx]

# Quick guide to PLS regression model using SIMCA®^[[1]](#footnote-1)^ software

The sections below outline how the authors conducted the PLS regression model on qualitative data using SIMCA software (formerly SIMCA-P). SIMCA is a versatile multivariate data analysis software using a graphical design.

## Import data and Prepare data (SIMCA Import)

- Upon opening SIMCA, start a new project. In this case, select ‘Regular project’. A project is a file including the results of the analyses of a dataset.
- Once the project is created, a new window appears to import the dataset. The dataset we imported was an Excel format file. The rows are the themes and the columns are the interviews.
- Once the dataset is imported,
  - Set the row as the primary variable ID (in our work, the first row with variable labels) and the column as the primary observation ID (in our work, the first column containing the theme identifier).
  - Define the data types: in our work, all our variables were quantitative.
  - Define the variable roles to prepare the PLS regression model: select the variables that are X-variable (in our work, the first set of interviews including the first three quarters) and variables that are Y-variable (in our work, the last set of interviews including the last quarter)
  - Verify the dataset and parameters selected for variables and observations. Once verified, click to ‘Finish import’ button.

## Build PLS regression model (Workset)

- Once the dataset is imported and prepared, create a new model and select the PLS regression model
- In the ‘Variable’ tab, verify that the X variables and Y variables are correct (in our work, out of the 12 interviews, the first nine interviews were the X and the last three interviews were the Y)
- In the ‘Observations’ tab, verify that the number of observations included is correct (in our work, the sample size was 67)
- In the ‘Finish’ tab, verify that your model specifications are correct (most of the specifications are those set by default):
  - Verify that the model type is a PLS model
  - Verify that the fit model uses the first two components at least (this can be decreased or increased afterwards)
  - Use of cross-validation with confidence level of 95% and significance level of 0.05.
  - Distance to model is normalized in units of standard deviation
  - The coefficients are scales and centered
  - The residuals are standardized and R^2^ is explained variation

## Generate outputs

In the ‘Home’ tab, SIMCA allows for generating various statistical outputs through lists or figures. Here below are the instructions to generate the outputs provided in the manuscript:

- ***Table 1: Cumulative percentage of variation of the first set of interviews and the last set of interviews taken into account by PLS factor*** is obtained once the model is specified. The number of PLS factors (components) can be set in the ‘Model’ command.
- ***Figure 3: Correlation-loading plot of the relationships between interviews overlaid with the themes in the 2 PLS factors space*** is generated in the ‘Diagnostics & interpretation’ command under the ‘Loadings’ button with drop-down. Open the ‘Loading scatter plot’ dialog and tick ‘Correlation scaled’.
- ***Figure 4: Projection of the themes in the space of the predictor score (t1) and the response variable scores (u1) according to the first PLS factor*** is generated in the ‘Diagnostics & interpretation’ command under the ‘Scores’ button with drop-down. Open the ‘Score scatter plot’ dialog and select t1 and u1 in the ‘Selected’ table.
- ***Figure 5: Projection of the themes in the space of the predictor score (t2) and the response variable scores (u2) according to the second PLS factor*** is generated in the ‘Diagnostics & interpretation’ command under the ‘Scores’ button with drop-down. Open the ‘Score scatter plot’ dialog and select t2 and u2 in the ‘Selected’ table.

Documentation on SIMCA can be found at the following URL: <https://umetrics.com/products/simca>

1. At the time of the research, the PLS model was conducted using SIMCA-P® v10.0.4. The quick guide uses SIMCA v16.0.2 [↑](#footnote-ref-1)
